# Mesenchymal Stem Cells as Therapeutics Agents: Quality and Environmental Regulatory Aspects

**DOI:** 10.1155/2016/9783408

**Published:** 2016-11-24

**Authors:** Patricia Galvez-Martin, Roger Sabata, Josep Verges, José L. Zugaza, Adolfina Ruiz, Beatriz Clares

**Affiliations:** ^1^Advanced Therapies Area, Bioibérica S.A., 08029 Barcelona, Spain; ^2^Department of Pharmacy and Pharmaceutical Technology, School of Pharmacy, University of Granada, 180171 Granada, Spain; ^3^Department of Genetics, Physical Anthropology and Animal Physiology, University of the Basque Country, 48940 Leioa, Spain; ^4^Achucarro Basque Center for Neuroscience, Bizkaia Science and Technology Park, Building No. 205, 48170 Zamudio, Spain; ^5^IKERBASQUE, Basque Foundation for Science, María Díaz de Haro 3, 48013 Bilbao, Spain

## Abstract

Mesenchymal stem cells (MSCs) are one of the main stem cells that have been used for advanced therapies and regenerative medicine. To carry out the translational clinical application of MSCs, their manufacturing and administration in human must be controlled; therefore they should be considered as medicine: stem cell-based medicinal products (SCMPs). The development of MSCs as SCMPs represents complicated therapeutics due to their extreme complex nature and rigorous regulatory oversights. The manufacturing process of MSCs needs to be addressed in clean environments in compliance with requirements of Good Manufacturing Practice (GMP). Facilities should maintain these GMP conditions according to international and national medicinal regulatory frameworks that introduce a number of specifications in order to produce MSCs as safe SCMPs. One of these important and complex requirements is the environmental monitoring. Although a number of environmental requirements are clearly defined, some others are provided as recommendations. In this review we aim to outline the current issues with regard to international guidelines which impact environmental monitoring in cleanrooms and clean areas for the manufacturing of MSCs.

## 1. Introduction

Mesenchymal stem cells (MSCs) hold considerable promise as a source of cells for novel therapies treating many serious diseases and injuries, including metabolic, degenerative, and inflammatory diseases, repair and regeneration of damaged tissues, and cancer. MSCs can be isolated from different tissues of the human body, expanded and/or differentiated* in vitro*, and subsequently processed and administered to patients as medicine or stem cell-based medicinal products (SCMPs). The scope of potential MSCs-based therapies has expanded in recent years due to advances in stem cell research focused in regenerative medicine. Currently several SCMPs with MSCs have been approved by the regulatory authorities in different countries.

The manufacturing of MSCs for translational clinical research should be performed with appropriate controls that ensure their safety and quality. In this context, new regulatory regimes for advanced and complex treatments such as cell therapies, tissue engineering, and gene therapies have grown substantially in importance in developing countries because they offer ground-breaking new opportunities for the treatment of disease and injury [1, 2]. These measures require laboratories to gain new knowledge of cell manufacturing and regulatory strategies because there are a number of factors that contribute to the product quality, such as starting materials, packaging materials, validated processes, personnel, procedures, equipment, and premises and environment [3, 4]. Any procedure related to clinical application of MSCs requires a strict control in the production facilities. This includes the manufacturing space, the storage warehouse for raw and finished product, and support laboratory areas [5]. All these organized according to Good Manufacturing Practice (GMP) for pharmaceutical manufacturers. Among all these requirements, environmental contamination assessment for the manufacturing of MSCs plays an important role in minimizing the risk of contamination by particles or microorganisms. Contamination of MSCs can cause adverse reactions in patients (e.g., fever, chills, infections, and irreversible septic shock) and even death. Therefore it will be necessary to standardize and validate all procedures and analytical techniques involved in its manufacture by the implementation of quality control programs [6]. An environmental monitoring program must be established in the therapy laboratory. This formal program should clearly stipulate and evaluate all circumstances involving the microbiological quality of the process and the MSCs [7]. The amount and type of evidence required for microbiological quality control should be defined according to different regulatory bodies, such as national Pharmacopeias, regulatory authorities, and the International Standards Organization (ISO). Each analytical technique must be validated to assure that the adopted procedure does not alter the method and consequently the result [4].

This review provides all the necessary requirements to manufacture MSCs as medicine in order to present themselves as a new therapeutic alternative.

The current state of legislation and methodology for the environmental control monitorization are described.

## 2. Environmental Monitoring

The processing of MSCs for use in cell therapy protocols requires a specific environment in which air quality is controlled, in order to minimize the risk of contamination of cells. To control air quality monitorization of viable and nonviable particles must be carried out throughout the whole process. In this field, a viable particle is a particle that contains one or more living microorganisms. A nonviable particle is a particle that does not contain a living microorganism.

The environmental monitoring should include a series of physical controls (concentration of particles in the air, flow of air, integrity of high efficiency particulate air (HEPA) filters, differential pressure, temperature, and relative humidity) and microbiological tests [7]. Other aspects should be also determined: places and the frequency of the sampling, a map of the installations on which sampling points can be recorded, the actions required when the alert and action levels are observed, and the personnel control. In short, the main objective is to develop and preserve a controlled environment that minimizes the risk contamination of MSCs, with special care to critical processes with higher level risk.

Regular monitoring of the environment, process, and finished product with MSCs must occur according to a written procedure and in line with the published written standards and guidelines [8]. This written procedure is known as the environmental monitoring program which is designed to routinely monitor particulates and microorganisms in critical areas and provides meaningful information on the quality of the aseptic processing environment as well as environmental trends of ancillary clean areas [9].

## 3. Regulatory Sources

For a descriptive overview of the regulatory authorities and documents the following classification is presented below. However, this is a difficult task by the range of different regulatory documents and standards [10]. To date regulatory and other concerned authorities have not been able to unify and standardize the criteria for manufacturing of MSCs as SCMPs worldwide. There are still some differences over specific issues.

### 3.1. US Food Drug Administration (FDA)

The Food and Drug Administration (FDA) publishes guidance documents (not mandatory) to provide general requirements for investigators from the US Code of Federal Regulations (CFR). CFR is a compilation of all published federal laws in USA. All food and drug related laws are contained in its Title 21. Within this, part 211 is as follows: “Current Good Manufacturing Practice for finished Pharmaceuticals” [11]. One of the most important FDA guidances related to environmental monitoring is the “Guidance for industry: Sterile Drug Products Produced by Aseptic Processing—Current Good Manufacturing Practice” (FDA-cGMP) [9].

### 3.2. European Good Manufacturing Practices (EU-GMP)

The body of European Union legislation in the pharmaceutical sector is compiled in the publication “The Rules Governing Medicinal Products in the European Union” published by the European Commission [12]. This consists of 10 volumes. Volume 4 contains guidance for the interpretation of the principles and guidelines of GMP for medicinal products for human and veterinary use.

The European Medicines Agency (EMA) is the responsible public body for the scientific evaluation of medicines. Important documents of this regulation are Volume 4, annex 1: “Manufacture of Sterile Medicinal Products” [13] and annex 2: “Manufacture of Biological Active Substances and Medicinal Products for Human Use” [14]. On the other hand, EMA issued the “Guideline on Scientific Requirements for the Environmental Risk Assessment of Gene Therapy Medicinal Products” [15].

### 3.3. World Health Organization (WHO)

The WHO was the first international organization who established detailed guidelines for GMP. GMP guidelines for biological products were approved in 1992 by both the WHO Expert Committee on Biological Standardization and the WHO Expert Committee on Specifications for Pharmaceutical Preparations [16]. This guidance contains different annexes which have been revised over the course of the years. One of the most important annexes is annex 6 “WHO-GMP for Sterile Pharmaceutical Products” [17]. Specific keys for the manufacture of sterile products are also described in order to minimize the risk of microbiological contamination, including viable and nonviable particles and pyrogens. Based on scientific developments and GMP, some technical requirements may be modified [18].

### 3.4. Pharmaceutical Inspection Convention and the Pharmaceutical Inspection Cooperation Scheme (PIC/S)

The Pharmaceutical Inspection Convention and Pharmaceutical Inspection Cooperation Scheme are two international bodies, made up of 46 representatives participating authorities from different countries with competencies in the field of GMP. The PIC/S aim to harmonize inspection procedures by developing common standards of GMP. They also aim to facilitate cooperation and contacts between the competent authorities, regional and international organizations, thereby increasing mutual trust. As GMP guide of interest for this article was that issued by PIC/S is the “Guide to GMP for Medicinal Products” PE 009 and revisions [19].

Originally, the PIC/S GMP guide (“PIC Basic Standards” of 1972) derived from the WHO-GMP guide. However, it was further adapted and expanded to satisfy the requirements of states taking part in PIC/S. In 1989, the EU adopted its own GMP guide. Since then the EU and PIC/S GMP guidelines have been developed in parallel but differ on small points such as expressions or references to Pharmacopeias.

### 3.5. International Standard Organization (ISO)

ISO is an independent, nongovernmental membership organization developer of voluntary international standards. Its main aim is to promote the development of worldwide harmonization of standards. ISO publishes numerous standards of relevance to pharmaceutical manufacturing, but not all of these standards are associated with GMP conditions. The most important GMP guide related to the topic at hand is the standard ISO 14644: “Cleanrooms and Associated Controlled Environments” and its series [20]. These standards are referenced both in EU-GMP and FDA-cGMP.

### 3.6. International Conference on Harmonization of Technical Requirements for Registration of Pharmaceuticals for Human Use (ICH)

Harmonization of regulatory requirements was pioneered by the European Union (formerly European Community) in the 1980s moved towards the development of a single market for pharmaceuticals. At the same time, bilateral meetings between Europe, Japan, and the USA took place. Finally, at the WHO Conference of Drug Regulatory Authorities, in Paris (1989), clear statements began to materialize.

It publishes quality and GMP documentation. Launched in 1990, ICH is a unique undertaking that brings together the drug regulatory authorities and the pharmaceutical industry of Europe, Japan, and the USA. Among others, an important document regarding environment monitoring is “Good Manufacturing Practice Guide for Active Pharmaceutical Ingredients Q7” [21]. Some guidelines have been assumed by EU-GMP and FDA-cGMP.

### 3.7. Pharmacopeias

The main international Pharmacopeias regarding this field are European Pharmacopeia (EP), Japanese Pharmacopeia (JP), and the United States Pharmacopeia (USP). Pharmacopeias issue some aspects with direct relevance mainly to sterility testing and other laboratory test methods. An example is the* Mycoplasma* testing [22–24].

### 3.8. Other Guidance Sources

Some countries possess national regulatory agencies that publish additional documents of guidance such as Australia, Canada, Japan, and Singapore. These agencies include Parenteral Drug Association (PDA), American Society for Testing and Materials (ASTM), Pharmaceutical Microbiology Interest Group (Pharmig), Pharmaceutical and Healthcare Sciences and Society (PHSS), IPSE (International Society for Pharmaceutical Engineering) [10].

## 4. Facilities to Translational Clinical Application

A MSCs production laboratory for clinical use must meet the minimum requirements for the product sterility manufacture. These facilities are called cleanrooms or clean areas. Environmental parameters such as size and number of airborne particulates, temperature, humidity, air pressure, airflow patterns (speed and direction), air motion, vibration, noise, viable (living) organisms, radiation, and lighting must be strictly controlled [25].

According to the degree of purity of air three different international standards have been proposed and only particle contamination is used for classification purposes.

### 4.1. Federal Standard 209

This standard was first published in 1963 in the USA entitled “Cleanroom and Work Station Controlled Environments” and posteriorly revised five times until 1992. Finally, it was canceled in 2001. The Federal Standard categorized cleanrooms in six general classes, depending on the particle count (particles per cubic foot) and size in *µ*m. When expressed in SI units, the numerical designation of the class is derived from the logarithm (base 10, with the mantissa truncated to a single decimal place) of the maximum allowable number of particles, 0.5 m and larger, per cubic meter of air. When expressed in English (US customary) units, the numerical designation of the class is derived from the maximum allowable number of particles, 0.5 m and larger, per cubic foot of air (Table 1). For alternative classes less clean than class M4.5, verification shall be performed by measurement in different particle size ranges. This standard was superseded by ISO standard. However, many organizations refused to change due to expensive costs and currently; it is commonly accepted in some facilities in the USA and Asia.

### 4.2. ISO

Cleanrooms are classified according to the air cleanliness. In the international domain, the ISO Technical Committee 209 decided to draft an international standard on these cleanrooms, whose mission was to establish the criteria that should govern the cleanrooms without making specific reference to a particular through the ISO 14644 series [20]. The first international standard was the ISO 14644-1 [26], which was slowly replacing the Federal Standard 209E ratings. It is based on metric measurements. ISO 14644-1 covers the classification of air cleanliness in cleanrooms and other controlled environments. ISO 14644-1 has been revised as a new, second-edition Draft International Standard (DIS), the ISO/DIS 14644-1.2 [27]. However, it is not yet adopted as an American National Standard until published as such. The classification of this standard is based solely on the concentration of suspended particles (Table 2). Moreover, the only particle populations that are considered for classification are the cumulative distribution based on thresholds (lower limit) from 0.1 to 5 *µ*m.

### 4.3. EU-GMP

Each manufacturing operation requires an appropriate level of environmental cleaning to minimize the risk of microbial contamination or particles in the product or materials being handled. EU-GMP, annex 1: “Manufacture of Sterile Medicinal Products of GMP” [13], details the new considerations to make in the production of advanced drug therapies products making control of the number of particles in the working environment of the cleanroom. For the manufacture of sterile medicinal products four grades can be distinguished: grade A in the local zone for high risk operations, grade B for aseptic preparation and filling operations (background environment of the grade A zone), and grades C and D for clean areas in which less critical stages are carried out in the manufacture of sterile products.

Two conditions are defined depending on the manufacturing activity: “*in operation*” and “*at rest*.” And thus different air-cleanliness levels must be specified. As the EU-GM itself defines, the “*at rest*” state is one in which the cleanroom is operational, with all the equipment and HVAC systems without staff present. On the other hand, in the “*in operation*” state the installation is in the operating mode with all staff, which will be previously defined [13].  Table 3 reports the airborne particulate classification for these grades, according to the PIC/S GMP and EU-GMP. There is a correspondence between these guidance conditions and that specified in the ISO 14644-1 at a particle size of 0.5 *μ*m.

To achieve the degree of air A, B, C, and D, the number of air changes should be related to the size of the room and the equipment and personnel present in it; the air system must have appropriate filters such as HEPA grades A, B, and C. The HEPA filter is not mentioned for grade D.

## 5. Methods for Environmental Monitoring of Cleanrooms

**Figure 1:**
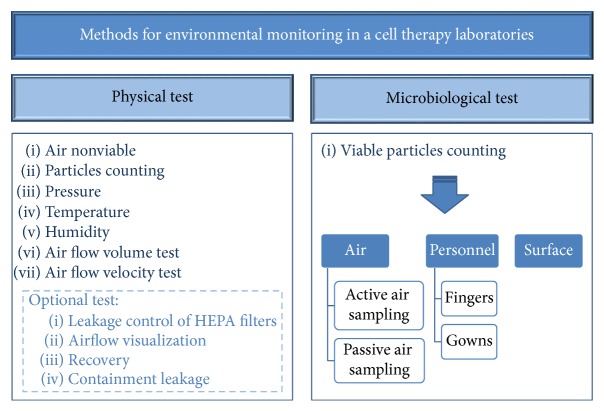
Scheme of environmental control requirements for the manufacture of SCMPs in cell therapy laboratories for the monitorization of viable and nonviable particles.

Airborne particles can be shaped and composed of different materials. They can also act as “carriers” for bacteria and other microorganisms. Hence, to distinguish between viable particles and inert particles (nonviable), analysis methods in a cleanroom can be classified as microbiological and physical tests. Microbiological tests consist of viable particles counting in both air and surfaces. Physical tests consist of air nonviable particles counting, pressure, and temperature analysis. Monitoring of both physical and microbiological contamination remains essential in aseptic operations to provide ongoing information on the maintenance of a stable and suitable environment for the aseptic preparation of products for administration to patients. It is vital that test methodologies exist as part of the environmental monitoring programme. Each test method selected for routine monitoring should be validated [8]. Techniques used for monitoring should be easy to perform, produce meaningful results, and must not contribute to contamination.  Figure 1 schematizes environmental control requirements for viable and nonviable particles.

ISO 14644 specifies basic requirements for cleanroom operations. This standard considers all classes of cleanrooms used to produce all types of products and does not address specific requirements for the pharmaceutical industry. A total of thirteen tests are described in this standard. However, only specific tests for cleanrooms intended for the production of SCMPs are commented on in the following sections. Some of them are mandatory but others are voluntary. The key controlling factors in the quality level of any cleanroom are the owner's requirements and what measurements are necessary to achieve that level of performance.

### 5.1. Frequency and Collection Sites

The frequency of environmental testing should have a direct relationship to the operations performed and be sufficient to allow for meaningful statistical calculations. FDA-cGMP, EU-GMP, USP, or ISO do not provide specific references for that issue but rather general recommendations as shown in Table 4. On the other hand, the WHO paper for manufacturers of human vaccines also provides indications in this respect [28].

**Figure 2:**
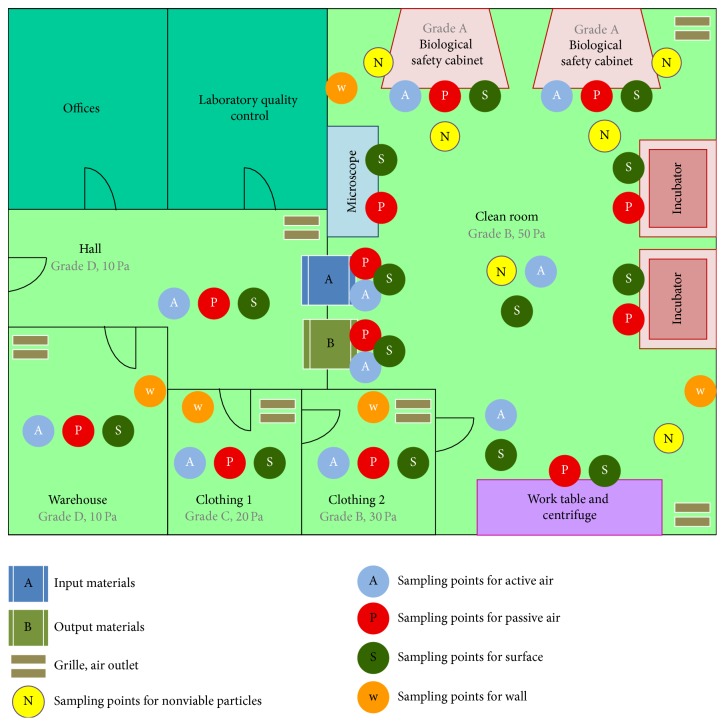
Diagram of cleanrooms and sampling points of environmental monitoring for stem cell units. Sampling N should be carried out whenever an activity is performed (in operation). The sampling rate for the points A, P, S, and W must be previously validated according to the requirements of the operations.

The minimum number of sampling point locations (NL, rounded up to a whole number) is defined by ISO 14644-1, annex B, through the following equation:

(1) $$\begin{array}{c} NL=\sqrt{A}, \end{array}$$

where *A* is the area of the cleanroom or clean zone in m^2^. In the case of unidirectional horizontal airflow, the area may be considered as the cross section of the moving air perpendicular to the direction of the airflow. Samples should be taken at approximately by dividing the clean area into a grid (one sample from each location) at 1 m above the floor approximately or at height of the work area. In the case of only one location, three samples are required. The required volume per sample depends on the cleanliness and the functional state of the environment. The minimal sample volume (*V*_*s*_, L) for qualification is established by annex B of the ISO 14644-1 guideline through the equation

(2) $$\begin{array}{c} V_{s}=\frac{20}{C_{n,m}}\times 1\text{,}000, \end{array}$$

where *C*_*n*,*m*_ is the class limit (number of particles per m^3^) for the largest considered particle size specified for the relevant class and 20 is the number of samples that could be counted if the particle concentration was at the class limit. The volume sampled at each location shall be at least 2 L, with the minimum sampling time at each location being 1 min. When *V*_*s*_ is very large, the time required for sampling can be substantial. In these cases the sequential sampling procedure described in annex F is followed, and both the required sample volume and time required to obtain samples may be reduced.  Figure 2 schematizes sampling points according to the clean area type. This type and the conditions will determine the frequency (Table 4).

## 6. Physical Tests

Measurement and determination of different physical operation aspects of the cleanroom are essential to ensure that a suitable environment is maintained for the preparation of aseptically prepared products.

### 6.1. Nonviable Particle Counts

For the measurement of particle concentration in grade A and B areas a continuous system should be used, with the establishment of the required frequency and alert limits. The volume of the air sample should not be less than 1 m^3^ in both areas and also in grade C areas.  Table 3 shows the maximum airborne particle concentration allowed in each area with light variations according to EU-GMP [12].

The locations of the monitoring systems of particles (according to risk analysis and classification results) should be next point to the product on display and working height, point of greater transfer of personnel and/or material, point on the remote environment of the area of influence of flow, and points with less effectively treated air flow (measured by the smoke test). Risk analysis is the quantitative or qualitative estimation of the likelihood associated with the previously identified hazards. A documented risk analysis to try to identify, evaluate, measure, and prevent possible failures that can initiate and trigger undesired events should be conducted by the manufacturer for ascertaining the appropriate GMP. Each cleanroom is different and therefore each of them should analyze all aspects related to the required environment. The risk analysis should consider all foreseeable hazards that may cause the input of pollutants. The location chosen for monitoring should be checked to ensure that the positions reflect the worst case. For room monitoring, the counts should be performed in locations where there is most operator activity. For the filling environment the counts should be performed adjacent to the filling zone and where components are exposed in such way as to detect operator activity within these areas.

Monitoring systems airborne particle counters may consist of independent particles, a network of sampling points for sequential access by a collector connected to a single particle counter, or a combination of both. The selected system must be appropriate to the particle size considered. It should be noted that sampling cannot compromise the laminar airflow in the critical zone and that the counting device is oriented in the direction of air flow input. It is standard practice to utilize modern technology and use an optical particle counter where the air sample is drawn into the instrument and passed through a light scattering device.

The terminology of ISO 14644-7 “Cleanrooms and Associate Controlled Environments” is “*separative devices*,” which includes laminar flow cabinets, minienvironments glove boxes, and isolators. These devices normally operate at EU-GMP Grade A/ISO Class 5. In Europe “*cabinet*” is the most common term to refer to “*hood*,” which is more typical in USA [29].

### 6.2. Pressure

Temperature and pressure devices are used to monitor the process. Automatic systems should be previously validated. The air pressure values will depend on the laboratory design, but a differential pressure from the most critical room to the outside of at least 30 Pa and 10–15 Pa between rooms is recommended [9, 30]. According to the ISO 14644-3, annex B5 pressure differential readings should be logged in all classes of cleanrooms in a maximal time interval of 12 months [31]. However, the interval between tests should be defined depending on the product and the process. Equally, recommendations regarding air supplies and pressure differentials may need to be modified depending on requirements [19]. A warning system and indicators of pressure with regular recording should be installed between areas.

### 6.3. Airflow Volume and Velocity

In grade A cleanrooms should be provided with laminar air flow with air speed of 0.36–0.54 m/s with regular validation [19]. Airflow volume test is intended to verify the air change rates by means of air flow readings and air change rates. It may be determined by either velocity or volume measurement techniques according to ISO 14644-3, annex B13 [32].

Verification laminar flow protection systems and the suitability of the containment conditions are performed to control the airflow velocity to be measured according to ISO 14644-3, annex B4 [33]. The acceptance criterion, according to EU-GMP and FDA-cGMP guidelines, is 0.45 m/s ± 20%.

Both tests should be performed in all cleanrooms at maximal period of twelve months as a reference in the operational and the at rest state. These tests could be performed by the installation of anemometers (direct air velocity measurement), manometers (indirect air velocity measurement), and pitot tube (single-point probe).

### 6.4. Optional Tests

Other optional tests such as installed filter leakage, airflow visualization, recovery, and containment leakage are defined in the ISO 14644-3 and suggest a retesting interval of 24 months.

#### 6.4.1. Installed Filter Leakage

Any air admitted should be passed through a HEPA filter [19]. HEPA filters are of paramount importance in obtaining viable and nonviable cleanliness levels, which retain particles greater than 0.3 *μ*m. Two filter integrity test methods for HEPA filters are reported in the ISO 14644-3, annex B6 [34]. Both methods require an evenly distributed aerosol challenge and the scanning of the filter gasket, filter frame, and filter media downstream of the filter.

The first* in situ* HEPA filter test method is DOP (dioctyl phthalate) test. This test utilizes the aerosol photometer as the measuring device and an aerosol generator to produce an aerosol challenge (scan testing). This method has been used since the 1950s and appears in many different standards such as MIL-STD-282 [35], IES RP-CC-001-83 [36], and European standard EN 1822-1 [37]. Now PAO (poly-alpha-olefin), diethylhexyl sebacate (DEHS), and paraffin are often used as aerosols. Sometimes the term DOP test is used to describe a HEPA filter leak test without intending to specify the use of DOP as the aerosol.

The second method offered in the standard is the particle counting method. This method also requires that the filter be evenly challenged with a known recorded concentration of aerosol, an aerosol diluter, and a discrete particle counter (volumetric testing). This procedure is implemented by using dissolution chambers and other devices that minimize the exposure of the delicate optical part of the device [38]. Results from both methods are not directly comparable. An unacceptable leak is defined as a penetration of 0.03% or more of particles 0.3 *µ*m and larger than the reference calibration curve for 99.97% efficient filters or as penetration of 0.01% or greater of particles 0.3 *µ*m and larger than reference calibration curve for 99.99% efficient filters [38].

#### 6.4.2. Airflow Visualization

This gives some idea as to how quickly contamination may be removed from the cleanroom provided that there is acceptable mixing of air in the room. An assessment of air flows (from clean to dirty areas) is a specification for the manufacture of sterile products, to evaluate ISO class 5 (Grade A zone) and the surrounding ISO class 7 (Grade B) room and uniformly from unidirectional air flow units. This is undertaken by visualizing actual or video-taped the air flow with the use of smoke in accordance with ISO 14644-3, annex B7 [39].

#### 6.4.3. Recovery

Also known as the clean-up time, recovery is the time elapsed in a cleanroom to return to the static condition (in terms of particulates), according to its classification, after an incident. In accordance with ISO 14644-3, annex B13 [32], it should not take more than 15 min. This standard contains two test methods known as cleanliness recovery performance and cleanliness recovery rate.

#### 6.4.4. Containment Leakage

It is designed to ensure that no airborne contamination can occur via leaks from higher pressure work areas to others adjacent to it. Airborne contamination can come into a cleanroom from less clean adjacent areas and pass through doors and hatches, as well as through holes and cracks in the walls, ceilings, and other parts of the cleanroom. In this way the absence of cross-contamination can be verified by the airflow direction smoke tests and room air pressures measurement in accordance to ISO 14644-3, annex B4 [33].

### 6.5. Less Critical Tests

A correct air quantity is necessary to displace particles, pressurize required spaces, and control temperature and humidity. This parameter is calculated as air changes per hour. According to ISO specifications it should be >120 air changes/h, >40 air changes/h, and >20 air changes/h for 100, 10,000, and 100,000 class cleanroom or clean area class, respectively. Airflow can also be used to determine the number of air changes that occur in a space over a period of one hour. This is accomplished by determining the supply (cm^3^/h) and dividing it by the total volume of a space (length × width × height) to come up with the number of air exchanges per hour.

Cleanrooms should have other requirements as temperature and humidity. These measurements will also assure the correct performance of the heating, ventilation, and air-conditioning (HVAC) system. However, some process steps require appropriate temperature. Moreover, the personnel commodity wearing special clothing should be taken into consideration. Relative humidity also affects occupant comfort, productivity, and operating costs. In general acceptance criteria are 22 ± 3°C (72 ± 5°F) temperature and 30–50% relative humidity.

On the other hand, the illuminance should be in accordance with the task to be performed. A range of 400 to 750 lux is recommended [10].

Finally, other physical tests for parameters as noise, vibration, or radiation have little or no applicability in cleanrooms for the processing of SCMPs.

## 7. Microbiological Tests

A major consideration in the operation of cleanroom technology for aseptic dispensing is the monitoring of viable contamination within clean environments [24]. Environmental monitoring is aimed to detect changing trends of microbial counts and microflora growth within the cleanroom [6, 40]. The results of the environmental monitoring provide information about the physical construction of the room, the performance of HVAC system [41], personnel cleanliness, gowning practices, and equipment and cleaning operations [42]. The microorganisms present in an environment will depend on the facilities, people, materials, equipment, processes, and environmental conditions of the area (temperature, humidity, presence of biocides, etc.). The most common potential forms of contamination in cell cultures are bacteria (including* Mycoplasma*), yeasts, and fungi, and these can be readily assessed on a routine basis [40].

The alert and action limits, expressed in cfu, should be established on the basis of levels of detection of microbial contamination. Action levels for nonviable particles are defined in the various regulatory and compendial documents for each room or area classification. Action levels are those that, when exceeded, indicate the appropriate corrective measure to return to the appropriate environmental safety. USA and European regulations, as well as, in the USP, chapter 1116, “Microbiological Control and Monitoring Environments Used for the Manufacture of Healthcare Products,” established the acceptable number of viable particles per m^3^ that can be found in determined cleanroom or clean area. WHO adopted the European standards. However, each company should set its own microbiological levels based on the aseptic requirements of it production. ISO does not refer to microbiological levels.

The methods used for microbiological monitoring include active air sampling (air sampler), passive air sampling (settle plates), surface sampling (contact plates and swabs), and personnel sampling (finger plates/plates of gowns). In order to carry out these operations the licensed manufacturer must be certified as a GMP manufacturer accredited by a recognized certification body in accordance with ISO 17025 or equivalent. However, it is not possible in Europe, where the GMP manufacturing is authorized by the national competent authority and recognized across the border on the basis of an international treaty. Currently, the US GMP authorization by FDA is not recognized in Europe and vice versa. The use of outside laboratories to carry out microbiological analysis can be accepted for particular reasons, as many companies are outsourcing technical testing activities and reducing in-house capabilities in an effort to control costs, but this should be stated in the quality control records. Manufacturers should use a risk-based approach to determine whether a preapproval audit is required before approving a contract laboratory. Various Agency guidance documents indicate how quality management principles relate to contract these operations. The ICH guidance for industry Q7 [21] recommends that manufacturers evaluate contractors for GMP compliance both by establishing a formal agreement that delineates GMP responsibilities, including quality measures, and by auditing the contractor's facilities [43].

### 7.1. Collection Sites and Frequency

The sampling plan for viable particles should define the number of points sampled in each of the areas of a cleanroom and determine how often to perform the sampling. According to the FDA-cGMP monitoring locations that present the highest potential contamination risk to the product and trending performance should be selected by assessing the critical activities taking place, the flow of personnel in the processing area, and the position of filters to determine the most potential high risk contamination locations. This approach is also stated in EU-GMP and ICH Q9 recommendations [12, 44]. Hazard Analysis Critical Control Points (HACCP) and Failure Mode Effect Analysis (FMEA) techniques are designed for this task. Sample locations for settle plates in cleanrooms should include those areas with the lowest air movement.

As discussed, the ISO 14644-1 guideline [26] provides a formula for the calculation of the minimum sampling locations for qualification of nonviable by dividing the area into a grid. Currently a randomly selection method using the grid is not recommended. Using a risk-based approach drives a continual review of trends and a periodic reassessment of the environmental programme.

Regarding the sampling frequency, it depends on the classification: the lower the maximum permitted particulate the higher the frequency of monitoring. GMP guidelines do not go into details. Table 4 shows recommendations of FDA-cGMP, EU-GMP, and USP. The reason why all these documents described only recommendation is because sample timing, frequency, and location should be carefully selected by the manufacturer based on the requirements of the operations performed and should be sufficient for allowing meaningful statistical calculations. Certain especial situations make necessary new microbiological testing such as corrective actions, after specifications changes, due to a change of activity or changes of environmental control equipment. Finally, when the specified microbial level of the cleanroom environment is exceeded, a documentation review and investigation should be carried out.

### 7.2. Microbial Growth Media

The selection of the growth media should assure the growth of microbes existing in the controlled environment. Thus, according to ISO 14698-1 [26], it is preferable to use a growth medium with low selectivity that is capable of supporting a broad spectrum of microorganisms including aerobes, anaerobes, fungi, and yeast, containing additive to overcome the residual effect of biocides and cleaning agents. The growth media should be validated thoroughly prior to using.  Table 5 lists EP recommendations for growth promotion test and the validation test. Specifications are similar to USP. The recommended size of solid media is 90 mm in diameter (approximate internal area 64 cm^2^) for settle plates and 55 mm (surface area 25 cm^2^) for contact plates. However, since 2012, FDA has permitted the use of alternative rapid microbiological methods.

Both USP and EP describe several adequate culture media for the sampling and quantification of microorganisms. As per USP Soybean Casein Digest Agar (SCDA) is the standard medium for sampling or quantitation of microorganisms in controlled environments. Yeasts and moulds may also be specifically sought out. Sabouraud Dextrose Agar is used especially for yeasts and moulds. As per EP fluid thioglycollate medium is primarily intended for the culture of anaerobic bacteria; however, it will also detect aerobic bacteria and SCDA for the culture of both fungi and aerobic bacteria.

For “settle plate” methods, Trypticase Soy Agar (TSA) is the most recommended medium for bacteria. It contains a mixture of peptones that promote the growth of most microorganisms. Agar Sabouraud Dextrose Chloramphenicol (SDC) is the recommended medium for fungi and yeast. Its high concentration of glucose optimizes the growth of fungi and its pH and chloramphenicol content improve the selectivity.

In order to choose the most efficient parameters for the test methodology, microbiologically, the best media and incubation conditions should be previously assayed, and parameters that yield the highest microbial recovery with the shortest incubation period are chosen for routine testing. Whether surfaces of testing were treated with detergents or disinfectant products, a neutralizing agent must be included in the recovery media. In this line, an antibiotic inactivating product must be incorporated in the recovery media if the testing surfaces have been treated with antibiotics.

### 7.3. Incubation Conditions

Total aerobic microbial count (TAMC) is determined by incubation in those media. The incubation conditions should be previously selected and validated. Culture conditions differ between microorganisms, 48 h at 32.5 ± 2.5°C for bacteria versus 72 h at 22.5 ± 2.5°C for fungi and moulds. Posteriorly, USP considered the possibility of longer incubation times. Equally, in case of absence of confirmatory evidence, one single plate may be incubated at both a low and a higher temperature. EP for its part recommends incubating the plates not more than 3 days in the case of bacteria at 30–35°C and not more than 5 days in the case of fungi at 20–25°C [45].

USP lists other permitted alternative media, liquid or solid. Furthermore, other alternative media to those listed can be used whether they are validated for the purpose intended.

### 7.4. Active Air Sampling Collection

Critical areas' monitoring should be carried out under “worst case” conditions for contamination with process equipment running and personnel performing normal operations (“*in operation*”) state [12]. Monitoring control should not interfere with critical work zone protection or compromise the quality of any products prepared that may be administered to patients. Measurements are performed as cfu per cubic meter of air (cfu/m^3^). All active air samplers work on the principle of sucking or blowing a stream of air at a sufficiently high velocity to cause any microorganisms in the sample to be impacted against a chosen medium. The two main types of equipment are the centrifugal and impaction devices. In all cases after the specified sampling time, the agar strip, plate, or filter in the sampler is removed, incubated under appropriate conditions, and then examined for microbiological growth. Preservation of the biological integrity and growth capacity of the microorganisms following impact are critical [46]. The sample size of air to be sampled is one of the main limitations of mechanical air samplers. The choice of an air sampler can be determined by the validation of the instrument, either by the manufacturer or a third party, in agreement with annex B of the ISO 14698-1 [26]. Recommended action limits for microbiological active monitoring of cleanrooms and clean areas are depicted in Table 6.

### 7.5. Passive Air Sampling Collection

Passive air or sedimentation sampling is based on the fact that, in absence of any kind of influence, airborne microorganisms which typically are attached to large particles will deposit onto open culture plates (settle plates) [47]. Thus, Petri dishes containing agar medium are opened and exposed in the cleanroom at working height for a specific time period (4 h to prevent media desiccation). Positive and negative controls should be also exposed. This method allows continuous sampling throughout a given work period, although they cannot indicate variation of contamination levels throughout the sampling period.

The cleanroom should be “*at rest*” to monitor baseline contamination levels. However, if the test conducted when operational, it will be affected by movements of the personnel and air flow. But it is considered a qualitative method and does not represent concentration of airborne microorganism. After incubation, results are reported as number of cfu per 4 h according to EU-GMP and FDA-cGMP. Recommended action limits for microbiological passive monitoring of cleanrooms and clean areas are depicted in Table 6.

### 7.6. Surfaces Sampling

EU-GMP [12] and USP [48] require surface monitoring of facilities (wall, floor, work surfaces, ceiling, etc.) furniture, equipment, and garment at the end of processing and after sanitation. Surfaces may become contaminated in a number of ways, for example, microorganisms settling out from the environment or from the direct touch by an operator [49]. One of the objectives of surface sampling is to determine the efficiency of routine cleaning procedures in removing contamination. The most frequent method is using contact plates. These are Petri dishes filled with appropriate growth medium and effective area of 25 cm^2^ according to EU-GMP or from 24 to 30 cm^2^ according to USP. Specially designed plates for this task are the RODAC (replicate organism detection and counting) plates commercially available with TSA or SDC with Lecithin and Polysorbate 80 added to inactivate residual disinfectants. Contact plates have a raised agar surface which is placed lightly onto the surface for 15 s and then incubated. After sampling collection with an agar-containing device, it should be cleaned with 70% alcohol to avoid the promotion of microbes.

Another contact method for surfaces where contact plates could not be utilized is to undertake a swabbing with sterile swabs. When swabbing is used in sampling, the area covered should be greater than or equal to 24 cm^2^ but no larger than 30 cm^2^ as stated by USP. After swabbing, the swab should be placed into a suitable culture medium or a diluent, vortexed for about 30 s, and then tested by pour-plate or membrane filtration method [50]. This method sampling should be used in areas with probability of contamination.

Finally, flexible films are reported by the PDA [8]. The media are deposited on a flexible substrate which can be used in an identical manner to that employed for contact plates. After incubation, results are reported as number of cfu per plate according to EU-GMP and USP. Recommended action limits for surface sampling monitoring of cleanrooms and clean areas are depicted in Table 7.

### 7.7. Personnel Sampling Monitoring

Only personnel who are qualified and appropriately gowned should be permitted access to the aseptic manufacturing area. Personnel can significantly affect the quality of the environment in which the product is processed; for this reason only the minimum number of personnel required should be present in cleanroom. Methods for personnel microbiological testing should include gloves and protection clothes at the end of each working session prior to the operator carrying out any cleaning or tidying operations. For this the desired area of the protection clothing is placed lightly onto the surface of the agar medium of the settle plate. On the other hand, the glove finger count checking is done randomly among individuals by finger dab plates in each of the five fingers of both hands. Finger dabs can be performed using either standard 90 mm diameter settle plates or 55 mm diameter contact plates. After incubation, results are reported as number of cfu per glove according to EU-GMP or cfu/plate according to FDA-cGMP. Recommended action limits for personnel sampling monitoring of cleanrooms and clean areas are depicted in Table 8.

## 8. Microorganism Identification

FDA-cGMP has clearly recommended the establishment of a listing of common microorganisms found in the aseptic manufacturing environment [9]. The identification of microorganisms to the species (or, where appropriate, genus) provides vital information for the environmental monitoring and for investigation. Some species are more prone to be promoted for human activity (*Staphylococcus*,* Micrococcus*). Contrary, other species are supposed to be related to environment (*Bacillus*,* Penicillium*, or* Pseudomonas*).

It is so important to have knowledge of the “normal” background flora of a cleanroom facility. Any unusual organisms or deviation from “normal” flora may require corrective actions.

## 9. Test Report

ISO 14644-1 includes the elaboration of a test report after testing in this way. The results from testing each cleanroom or clean zone shall be recorded and submitted as a comprehensive report, along with a statement of compliance or noncompliance with the specified designation of airborne particulate cleanliness classification. This standard provides for the inclusion, among other information, physical description of facilities, designation criteria for the cleanroom or clean zone, test methods, and test results.

## 10. Conclusions

The field of MSCs manufacture includes the task of interpreting and harmonizing international guidelines to ensure their acceptable quality for translational clinical use in regenerative medicine. One of the great challenges for the future is to set a single regulatory framework for the SCMPs manufacture, through harmonization of all the requirements for their production whatever their use or intended final purpose: gene therapy, cell therapy, or tissue engineering or whenever their production: USA, Europe, Japan, and so forth.

## Figures and Tables

**Table 1:** Federal Standard 209E. Class limits are given for each class name.

Class name		Class limits									
		≥0.1 *µ*m		≥0.2 *µ*m		≥0.3 *µ*m		≥0.5 *µ*m		≥5 *µ*m	
SI	English	m^3^	ft^3^	m^3^	ft^3^	m^3^	ft^3^	m^3^	ft^3^	m^3^	ft^3^
M1		350	9.91	75.7	2.14	30.9	0.875	10.0	0.283		
M1.5	1	1,240	35	265	7.50	106	3.00	35.3	1.00		
M2		3,500	99.1	757	21.4	309	8.75	100	2.83		
M2.5	10	12,400	350	2,650	75.0	1,060	30.0	353	10.0		
M3		35,000	991	7,570	214	3,090	87.5	1,000	28.3		
M3.5	100			26,500	750	10,600	300	3,530	100		
M4				75,700	2,140	30,900	875	10,000	283		
M4.5	1,000							35,300	1,000	247	7.00
M5								100,000	2,830	618	17.5
M5.5	10,000							353,000	10,000	2,470	70.0
M6								1,000,000	28,300	6,180	175
M6.5	100,000							3,350,000	100,000	24,700	700
M7								10,000,000	283,000	61,800	1,750

**Table 2:** ISO-14664, cleanrooms, and associated controlled environments (particles/m^3^).

ISO classification number (*N*)	Class limits					
	≥0.1 *µ*m	≥0.2 *µ*m	≥0.3 *µ*m	≥0.5 *µ*m	≥1.0 *µ*m	≥5.0 *µ*m
1	10	2				
2	100	24	10	4		
3	1,000	237	102	35	8	
4	10,000	2,370	1,020	352	83	
5	100,000	23,700	10,200	3,520	832	29
6	1,000,000	237,000	102,000	352,000	8,320	293
7				3,520,000	83,200	2,930
8				35,200,000	832,000	29,300
9					8,320,000	293,000

**Table 3:** Airborne particulate classification for these grades, according to the PIC/S GMP and EU-GMP.

Grade	Maximum number of particles permitted/m^3^			
	At rest		In operation	
	≥0.5 *µ*m	≥5.0 *µ*m	≥0.5 *µ*m	≥5.0 *µ*m
A^a^	3,520	20	3,520	20
B	3,520	29	352,000	2,900
C	352,000	2,900	3,520,000	29,000
D	3,520,000	29,000	Not defined	Not defined

^a^All areas must be free particles of size greater than 5 *μ*m. Limits are set to 1 particle/m^3^ because it is impossible to ensure the absence of particles with any statistical significance. The periodic classification of facilities (cleanroom) must show that all areas meet the defined limits.

**Table 4:** Recommended frequency of environmental monitoring testing.

Clean area type			Frequency sampling			
			FDA-cGMP	EU-GMP		USP
FS209E	ISO	EU-GMP		At rest	In operation	
M3.5 (100)	5	A	Should cover all production shifts	Frequent to detect system deterioration	For the duration of critical operations	Each operating shift
M4.5 (1,000)	6	—^a^	Should cover all production shifts	—	—	Each operating shift
M5.5 (10,000)	7	B	Should cover all production shifts	Frequent to detect system deterioration	For the duration of critical operations	Each operating shift
M6.5 (100,000)	8	C	Should cover all production shifts	Frequent to detect system deterioration	In line with quality risk management	Twice a week Once a week^b^
		D	—	In line with quality risk management	In line with quality risk management	—

^a^There is no correspondence between FS209 M3.5 (100) and ISO 6 classes with EU-GMP cleanroom classification.

^b^Other support areas to aseptic processing areas but nonproduct contact.

**Table 5:** Strains of the test microorganisms suitable for use in the growth promotion test and the validation test.

Microorganism		Strains
Aerobic	*Staphylococcus aureus*	ATCC 6538, CIP 4.83, NCTC 10788, NCIMB 9518
	*Bacillus subtilis*	ATCC 6633, CIP 52.62, NCIMB 8054
	*Pseudomonas aeruginosa*	ATCC 9027, NCIMB 8626, CIP 82.118
Anaerobic	*Clostridium sporogenes*	ATCC 19404, CIP 79.3, NCTC 532 or ATCC 11437
Fungi	*Candida albicans*	ATCC 10231, IP 48.72, NCPF 3179
	*Aspergillus niger*	ATCC 16404, IP 1431.83, IMI 149007

**Table 6:** Recommended limits for viable airborne particles in the environment according to FDA-cGMP, EU-GMP, and USP.

Clean area type			Maximal number of cfu in the environment				
			FDA-cGMP		EU-GMP		USP
FS209E	ISO	EU-GMP	Air sample (cfu/m^3^)	Settle plates^a^ (diam. 90 mm; cfu/4 h)	Air sample (cfu/m^3^)	Settle plates (diam. 90 mm; cfu/4 h)^b^	Air sample (cfu/m^3^)
M3.5 (100)	5	A	1^c^	1^c^	<1	<1	<3
M4.5 (1,000)	6		7	3	—	—	
M5.5 (10,000)	7	B	10	5	10	5	<20
M6.5 (100,000)	8	C	100	50	100	50	<100
		D	—	—	200	100	—

^a^The additional use of settling plates is optional.

^b^Individual settle plates may be exposed for less than 4 hours.

^c^Samples from class 100 (ISO 5) environments should normally yield no microbiological contaminants.

**Table 7:** Recommended limits for viable airborne particles on surfaces according to EU-GMP and USP.

Clean area type			Maximal number of cfu on surfaces		
			EU GMP	USP	
FS209E	ISO	EU GMP	Contact plates (diam. 55 mm; cfu/plate)	Contact plates (area 24–30 cm^2^; cfu/plate)^a^	
				Surfaces	Floor
M3.5 (100)	5	A	<1	3	3
M4.5 (1,000)	6		—	—	—
M5.5 (10,000)	7	B	5	5	10
M6.5 (100,000)	8	C	25	—	—
		D	50	—	—

^a^Contact plate areas vary from 24 to 30 cm^2^. When swabbing is used in sampling, the area covered should be greater than or equal to 24 cm^2^ but no larger than 30 cm^2^.

**Table 8:** Recommended limits for viable airborne particles on personnel according to FDA-cGMP, EU-GMP, and USP.

Clean area type			Maximal number of cfu		
			EU-cGMP	USP	
FS209E	ISO	EU GMP	Glove print (5 fingers) (cfu/glove)	cfu per contact plate	
				Gloves	Personnel clothing and garb
M3.5 (100)	5	A	<1	3	5
M5.5 (10,000)	7	B	5	10	20
